# Supplementary material for: A calcineurin-mediated scaling mechanism that controls a K+-leak channel to regulate morphogen and growth factor transcription
Source: eLife. 2021 Apr 8;10:e60691. doi: 10.7554/eLife.60691 (PMC8110307; doi:10.7554/eLife.60691)
Supplement: Transparent reporting form [file elife-60691-transrepform.pdf]

1st Floor
24 Hills Road
Cambridge CB2 1JP, UK

P 01223 855340
W elifesciences.org
T @elife

eLife's transparent reporting form

We encourage authors to provide detailed information *within their submission* to facilitate the interpretation and replication of experiments. Authors can upload supporting documentation to indicate the use of appropriate reporting guidelines for health-related research (see EQUATOR Network), life science research (see the BioSharing Information Resource), or the ARRIVE guidelines for reporting work involving animal research. Where applicable, authors should refer to any relevant reporting standards documents in this form.

If you have any questions, please consult our Journal Policies and/or contact us: editorial@elifesciences.org.

Sample-size estimation

- You should state whether an appropriate sample size was computed when the study was being designed
- You should state the statistical method of sample size computation and any required assumptions
- If no explicit power analysis was used, you should describe how you decided what sample (replicate) size (number) to use

Please outline where this information can be found within the submission (e.g., sections or figure legends), or explain why this information doesn't apply to your submission:

For animal experiments, we determined numbers a priori based on estimated differences between control and experimental groups from our previously published findings (Kujawski et al, 2014) by using statistical prediction software (G-power) http://www.psycho.uni"duesseldorf.de/aap/projects/gpower. We predicted the number of animals necessary to be able to observe at least a $P<0.05$ value using the two-paired students' T-test as a determination of statistical significance.

For in vitro experiments, no power analysis was used. We performed three or four experiments with two or more replicates and evaluated the statistical significance post hoc using the two-paired students' T-test.

Replicates

- You should report how often each experiment was performed
- You should include a definition of biological versus technical replication
- The data obtained should be provided and sufficient information should be provided to indicate the number of independent biological and/or technical replicates
- If you encountered any outliers, you should describe how these were handled
- Criteria for exclusion/inclusion of data should be clearly stated
- High-throughput sequence data should be uploaded before submission, with a private link for reviewers provided (these are available from both GEO and ArrayExpress)

Please outline where this information can be found within the submission (e.g., sections or figure legends), or explain why this information doesn't apply to your submission:

eLife Sciences Publications, Ltd is a limited liability non-profit non-stock corporation incorporated in the State of Delaware, USA, with company number 5030732, and is registered in the UK with company number FC030576 and branch number BR015634 at the address 1st Floor, 24 Hills Road, Cambridge CB2 1JP | August 2014

The number of experiments and their replicates are described in the figure legends for each experiment. We excluded electrophysiological reads when we did not get complete patches (loss of pipette-cell membrane seal) or cells did not survive the patch clamp. We did not exclude any other data points. Each data point from the in vivo experiments were biological replicates from individual fish. For the in vitro cultured cells, at least two biological replicates (n) were used for each separate experiment (N), and each biological replicate was determined from at least two technical replicates.

Statistical reporting

- Statistical analysis methods should be described and justified
- Raw data should be presented in figures whenever informative to do so (typically when N per group is less than 10)
- For each experiment, you should identify the statistical tests used, exact values of N, definitions of center, methods of multiple test correction, and dispersion and precision measures (e.g., mean, median, SD, SEM, confidence intervals; and, for the major substantive results, a measure of effect size (e.g., Pearson's r, Cohen's d)
- Report exact p-values wherever possible alongside the summary statistics and 95% confidence intervals. These should be reported for all key questions and not only when the p-value is less than 0.05.

Please outline where this information can be found within the submission (e.g., sections or figure legends), or explain why this information doesn't apply to your submission:

We provide this information in the each figure panel itself and/or in the figure legends of the related experiments. We describe how we portray error/derivation and state the statistical analyses used in the figure legends of the associated experiments. In general, we used SEM, ANOVA and T-tests for the electrophysiology data sets, and standard deviation, ANOVA or T-tests for the gene expression data sets. Significance values between groups are embedded in the figures to clearly link each significance value with compared experimental groups.

(For large datasets, or papers with a very large number of statistical tests, you may upload a single table file with tests, Ns, etc., with reference to sections in the manuscript.)

Group allocation

- Indicate how samples were allocated into experimental groups (in the case of clinical studies, please specify allocation to treatment method); if randomization was used, please also state if restricted randomization was applied
- Indicate if masking was used during group allocation, data collection and/or data analysis

Please outline where this information can be found within the submission (e.g., sections or figure legends), or explain why this information doesn't apply to your submission:

Masking was used once for the first biological replicate in the experiments provided in Figure 5B, C and Figure 6E-I, in which the experiment was set up by one person and measured by a different person. In these instances, the samples were given randomized call numbers and unmasked after the measurements. All other experiments were set up and measured by one person, so the experiments were not masked.

Additional data files ("source data")

- We encourage you to upload relevant additional data files, such as numerical data that are represented as a graph in a figure, or as a summary table
- Where provided, these should be in the most useful format, and they can be uploaded as “Source data” files linked to a main figure or table
- Include model definition files including the full list of parameters used
- Include code used for data analysis (e.g., R, MatLab)
- Avoid stating that data files are “available upon request”

Please indicate the figures or tables for which source data files have been provided:

The additional data files include all graphed data that is shown as averaged values. They are organized by their corresponding figure call. This includes the numbers of cells counted for the autonomy data, the fin length and body length measurements as well as the individual original electrophysiology measurements and their corresponding standardized conversion for the electrophysiology graphs are provided as MS Excel files.

eLife Sciences Publications, Ltd is a limited liability non-profit non-stock corporation incorporated in the State of Delaware, USA, with company number 5030732, and is registered in the UK with company number FC030576 and branch number BR015634 at the address 1st Floor, 24 Hills Road, Cambridge CB2 1JP | August 2014
